# An enhanced estimator of finite population variance using two auxiliary variables under simple random sampling

**DOI:** 10.1038/s41598-023-44169-5

**Published:** 2023-12-05

**Authors:** Sohaib Ahmad, Nitesh Kumar Adichwal, Muhammad Aamir, Javid Shabbir, Najwan Alsadat, Mohammed Elgarhy, Hijaz Ahmad

**Affiliations:** 1 Department of Statistics, Abdul Wali Khan University Mardan, Mardan, Pakistan; 2 School of Business, University of Petroleum and Energy Studies, Dehradun, India; 3 Department of Statistics, University of Wah at Wah Cant, Wah, Pakistan; 4 Department of Quantitative Analysis, College of Business Administration, King Saud University, P.O.Box 71115, 11587 Riyadh, Saudi Arabia; 5 Mathematics and Computer Science Department, Faculty of Science, Beni-Suef University, Beni-Suef, 62521 Egypt; 6 Section of Mathematics, International Telematic University Uninettuno, Corso Vittorio Emanuele II, 39, 00186 Rome, Italy; 7 Operational Research Center in Healthcare, Near East University, TRNC Mersin 10, 99138 Nicosia, Turkey; 8 Center for Applied Mathematics and Bioinformatics, Gulf University for Science and Technology, Mishref, Kuwait; 9 Department of Computer Science and Mathematics, Lebanese American University, Beirut, Lebanon

**Keywords:** Applied mathematics, Statistics

## Abstract

In this article, we have suggested a new improved estimator for estimation of finite population variance under simple random sampling. We use two auxiliary variables to improve the efficiency of estimator. The numerical expressions for the bias and mean square error are derived up to the first order approximation. To evaluate the efficiency of the new estimator, we conduct a numerical study using four real data sets and a simulation study. The result shows that the suggested estimator has a minimum mean square error and higher percentage relative efficiency as compared to all the existing estimators. These findings demonstrate the significance of our suggested estimator and highlight its potential applications in various fields. Theoretical and numerical analyses show that our suggested estimator outperforms all existing estimators in terms of efficiency. This demonstrates the practical value of incorporating auxiliary variables into the estimation process and the potential for future research in this area.

## Introduction

The use of auxiliary information can increase the precision of population parameter estimates in survey sampling, assuming that it is strongly associated with the study variable. To get precise estimates of the unknown population characteristics, auxiliary information is essential in parameter selection and at estimation stage. Estimating population variance is specifically important in populations that are likely to be skewed, and the accuracy of the estimation has been the focus of many research studies. By reducing the dispersion of the estimate obtained from various samples, we can obtain a more accurate representation of the population. Variance is a feature that can be used to describe a statistical population, and brilliant efforts have been made to estimate this characteristic as accurately as possible. The goal is to reduce the scattering of the calculated values, and appropriate use of auxiliary variables in survey sampling results in significant decrease in the variance of the estimator of unknown population parameter(s). In many situations, it is necessary to estimate the population variance of the study variable. The variance estimate for the population is crucial in a number of disciplines, such as agriculture, health, biology, and business, where we confront populations that are likely to be skewed. Everywhere in the world, there are differences in the things we do every day. The idea that no two things or persons are exactly alike is one that is widely held. For instance, a farmer must be well aware of how the geographical climate varies over time in order to decide how to sow his land. A clinician must have a thorough understanding of the variations in human hypertension and fever in order to administer the appropriate care.

The estimation of population variance has been a matter of interest for several researchers over the years. Garcia and Cebrian^1^ suggested the ratio estimator for the population variance. Upadhyaya et al.^2^ recommended a broad class of estimators for variance of the ratio estimator. Chandra and Singh^3^ discussed a family of estimators for population variance. Arcos et al.^4^ suggested incorporating the auxiliary information available in variance estimation. Kadilar and Cingi^5^ discussed improvement in variance estimation in simple random sampling. Grover^6^ discussed a correction note on improvement in variance using auxiliary information. Sharma and Singh^7^ discussed a generalized class of estimators for finite population variance. Singh and Solanki^8^ suggested improved estimation of finite population variance using auxiliary information. Yadav and Kadilar^9^ recommended a two parameter variance estimator using auxiliary information. Adichwal et al.^10^ proposed generalized class of estimators for population variance using auxiliary attribute. Adichwal et al.^11^ recommended generalized class of estimators for population variance using information on two auxiliary variables. Lone and Tailor^12^ suggested estimation of population variance in simple random sampling. Singh and Khalid^13^ discussed effective estimation strategy of population variance in two-phase successive sampling under random non-response. Audu et al.^14^ proposed difference-cum-ratio estimators for estimating finite population coefficient of variation in simple random sampling. Shahzad et al.^15^ suggested a new variance estimators for calibration approach under stratified random sampling. Singh and Khalid^16^ suggested a composite class of estimators to deal with the issue of variance estimation under the situations of random non-response in two-occasion successive sampling. Zaman and Bulut^17^ suggested a new class of robust ratio estimators for finite population variance. Shahzad et al.^18^ discussed use of calibration constraints and linear moments for variance estimation under stratified adaptive cluster sampling. Ashutosh et al.^19^ suggested calibration Approach for Variance Estimation of Small Domain. Ahmad^20^ discussed improved estimation of population variance under stratified random sampling. Ahmad^21^ discussed improved variance estimator using dual auxiliary variable under simple random sampling. Ahmad et al.^22^ suggested enhanced generalized class of estimators under simple random sampling.

Numerous authors have contributed to the estimation of population variance as mentioned above. In this article, we have suggested an enhanced estimator, which has valuable contribution and highlighted the importance of auxiliary variables in estimating of population variance. By using actual data and a simulation study, the suggested estimator has minimum mean square error and higher percentage relative efficiency as compared to recent well known existing estimators.

The primary goal of the current work is given by:

1. To propose a new estimator for finite population variance using two auxiliary variables under simple random sampling.
2. The properties i.e. bias and mean squared error of the propose estimator is derived up to the first order of approximation.
3. Through the use of the actual data and a simulation study, the application of the propose estimator is highlighted.
4. A comparison of estimators is done with existing estimators in terms of minimum mean square error.
5. Our proposed estimator provides a novel and valuable contribution to the field of population variance estimation, and we believe it has the potential to be useful in a wide range of applications.

## Notations and symbols

Consider $$\raisebox{2.1pt}{$\upomega$}\kern-2.3pt\rotatebox{80}{\tiny $\smile$}$$= $$\left({{\raisebox{2.1pt}{$\upomega$}\kern-2.3pt\rotatebox{80}{\tiny $\smile$}}}_{1},{{\raisebox{2.1pt}{$\upomega$}\kern-2.3pt\rotatebox{80}{\tiny $\smile$}}}_{2},\dots ,{{\raisebox{2.1pt}{$\upomega$}\kern-2.3pt\rotatebox{80}{\tiny $\smile$}}}_{\mathrm{N}}\right)$$ is a population comprised of size *N*, consider a sample of size *n* is chosen from $${\raisebox{2.1pt}{$\upomega$}\kern-2.3pt\rotatebox{80}{\tiny $\smile$}}$$ by using simple random sampling without replacement (SRSWOR). Let *Y* be the population study variable, and $${X}_{1}$$ and $${X}_{2}$$ denote the two auxiliary variables respectively. Consider population variance of *Y,* $${X}_{1}$$, and $${X}_{2}$$ are denoted by $${S}_{y}^{2}$$, $${S}_{x1}^{2}$$, and $${S}_{x2}^{2}$$. Let $${s}_{y}^{2}$$ = $$\frac{{\sum_{i=1}^{n}\left({y}_{i}-\overline{y }\right)}^{2}}{n-1}$$, $${s}_{x1}^{2}$$ = $$\frac{{\sum_{i=1}^{n}\left({x}_{1i}-{\overline{x} }_{1}\right)}^{2}}{n-1}$$, $${s}_{x2}^{2}$$ = $$\frac{{\sum_{i=1}^{n}\left({x}_{2i}-{\overline{x} }_{2}\right)}^{2}}{n-1}$$, be the sample variances. $${S}_{y}^{2}$$ = $$\frac{{\sum_{i=1}^{N}({y}_{i}-\overline{Y })}^{2}}{N-1}$$, $${S}_{x}^{2}$$ = $$\frac{{\sum_{i=1}^{N}\left({x}_{1i}-{\overline{X} }_{1}\right)}^{2}}{N-1}$$, $${S}_{x2}^{2}$$ = $$\frac{{\sum_{i=1}^{N}\left({x}_{2i}-{\overline{X} }_{2}\right)}^{2}}{N-1}$$, be the population variances. Let $$\overline{y }$$, $${\overline{x} }_{1}$$, and $${\overline{x} }_{2}$$ be the sample mean.

To derive the bias and mean square error, we consider the following error terms:

$${\xi }_{o}=\frac{{s}_{y}^{2}-{S}_{y}^{2}}{{S}_{y}^{2}},{\xi }_{1}=\frac{{s}_{x1}^{2}-{S}_{x1}^{2}}{{S}_{x1}^{2}},{\xi }_{2}=\frac{{s}_{x2}^{2}-{S}_{x2}^{2}}{{S}_{x2}^{2}},\mathrm{E}\left({\xi }_{i}\right)=0,\mathrm{for }(i=0, 1, 2).$$

$$E\left({\xi }_{o}^{2}\right)={\reflectbox{$\lambda$}}{{\mkern0.75mu\mathchar '26\mkern -9.75mu\lambda}}_{400}^{*},E\left({\xi }_{1}^{2}\right)={\reflectbox{$\lambda$}}{{\mkern0.75mu\mathchar '26\mkern -9.75mu\lambda}}_{040}^{*},E\left({\xi }_{2}^{2}\right)={\reflectbox{$\lambda$}}{{\mkern0.75mu\mathchar '26\mkern -9.75mu\lambda}}_{004}^{*},E\left({\xi }_{o}{\xi }_{1}\right)={\reflectbox{$\lambda$}}{{\mkern0.75mu\mathchar '26\mkern -9.75mu\lambda}}_{220}^{*},E\left({\xi }_{0}{\xi }_{2}\right)={\reflectbox{$\lambda$}}{{\mkern0.75mu\mathchar '26\mkern -9.75mu\lambda}}_{202}^{*},E\left({\xi }_{1}{\xi }_{2}\right)={\reflectbox{$\lambda$}}{{\mkern0.75mu\mathchar '26\mkern -9.75mu\lambda}}_{022}^{*},$$

$${{\mkern0.75mu\mathchar '26\mkern -9.75mu\lambda}}_{lmn}=\frac{{\upupsilon }_{lmn}}{{\upupsilon }_{200}^\frac{l}{2}{\upupsilon }_{020}^\frac{m}{2}{\upupsilon }_{002}^\frac{n}{2}},{\upupsilon }_{lmn}=\frac{\sum ({{y}_{i}-\overline{Y })}^{l}({{x}_{1i}-{\overline{X} }_{1})}^{m}{({x}_{2i}-{\overline{X} }_{2})}^{n}}{N-1}.$$

where $${{\mkern0.75mu\mathchar '26\mkern -9.75mu\lambda}}_{lmn}^{*}$$ =$$\left({{\mkern0.75mu\mathchar '26\mkern -9.75mu\lambda}}_{lmn} - 1\right)$$, and $${\reflectbox{$\lambda$}}$$=$$\left(\frac{1}{n}-\frac{1}{N}\right)$$.where *l*, *m*, and *n* be the positive numerals and $${\upupsilon }_{200}, {\upupsilon }_{020}$$, $${\mathrm{and \upsilon }}_{002}$$ are the second-order moments about means of *Y*, $${X}_{1}$$, and $${X}_{2}$$, and $${{\mkern0.75mu\mathchar '26\mkern -9.75mu\lambda}}_{lmn}$$ is the moment ratio.

The article has been structured as follows:

In Section "Introduction", we have provided a brief overview of the topic and highlighted the importance of estimating population variance. In Section “Notations and symbols”, we review some of the existing estimators for estimation of population variance. In Section "Existing estimators", we proposed a new estimator for population variance under simple random sampling. In Section "Proposed estimator", we will compare the proposed estimator with the existing estimators in terms of MSE. In Section "Efficiency assessment", we will present a numerical study to illustrate the performance of the proposed estimator and compare it with the existing estimators. In Section "Numerical study", we will perform simulations to validate the results of the numerical study and provide additional insights into the performance of the estimators. In Section "Simulation study", we will discuss the results of our study, the limitations of the proposed estimator, and the scope for future research in this field. In Section "Discussion", we will summarize the main findings of our study and provide concluding remarks on the estimation of population variance under simple random sampling.

## Existing estimators

In this section, we study different variance estimators based on simple random sampling that are available in the literature.

(i) The usual estimator ${\mathrm{T}}_{0}$, is given by:
  1 $${\mathrm{T}}_{0}=\frac{{\sum_{i=1}^{n}\left({y}_{i}-\overline{Y }\right)}^{2}}{n-1}$$
  The variance of ${\mathrm{T}}_{0}$, is given by:
  2 $$\mathrm{Var}\left({\mathrm{T}}_{0}\right)={\reflectbox{$\lambda$}}{S}_{y}^{4}{{\mkern0.75mu\mathchar '26\mkern -9.75mu\lambda}}_{400}^{*}$$
(ii) Isaki^23^ suggested a ratio estimator, which is given by:
  3 $${\mathrm{T}}_{1}={s}_{y}^{2}\left(\frac{{S}_{x}^{2}}{{s}_{x}^{2}}\right)$$
  The bias and mean square error of ${\mathrm{T}}_{1}$ are given as:
  $$\mathrm{Bias}\left({\mathrm{T}}_{1}\right)={\reflectbox{$\lambda$}}{S}_{y}^{2}\left\{{{\mkern0.75mu\mathchar '26\mkern -9.75mu\lambda}}_{040}^{*}-{{\mkern0.75mu\mathchar '26\mkern -9.75mu\lambda}}_{220}^{*}\right\},$$
  and
  4 $$\mathrm{MSE}\left({\mathrm{T}}_{1}\right)\cong {\reflectbox{$\lambda$}}{S}_{y}^{4}\left\{{{\mkern0.75mu\mathchar '26\mkern -9.75mu\lambda}}_{400}^{*}+{{\mkern0.75mu\mathchar '26\mkern -9.75mu\lambda}}_{040}^{*}-2{{\mkern0.75mu\mathchar '26\mkern -9.75mu\lambda}}_{220}^{*}\right\}.$$
(iii) The difference type estimator is given by:
  5 $${\mathrm{T}}_{2}={s}_{y}^{2}+\mathrm{k}\left({S}_{x}^{2}-{s}_{x}^{2}\right).$$
  where $\mathrm{k}$ is the constant, i.e. $\mathrm{k}$  = $\left[\frac{{S}_{y}^{2}{{\mkern0.75mu\mathchar '26\mkern -9.75mu\lambda}}_{220}^{*}}{{S}_{x}^{2}{{\mkern0.75mu\mathchar '26\mkern -9.75mu\lambda}}_{040}^{*}}\right]$.
  The minimum variance at the optimal value of $\mathrm{k}$ is given by:
  6 $$\mathrm{Var}\left({\mathrm{T}}_{2}\right)={S}_{y}^{4}{{\reflectbox{$\lambda$}}{\mkern0.75mu\mathchar '26\mkern -9.75mu\lambda}}_{400}^{*}\left(1-{\rho }^{2}\right),$$
  where $$\rho$$ = $$\frac{{{\mkern0.75mu\mathchar '26\mkern -9.75mu\lambda}}_{220}^{*}}{{\left({{\mkern0.75mu\mathchar '26\mkern -9.75mu\lambda}}_{400}^{*}\right)}^\frac{1}{2}{\left({{\mkern0.75mu\mathchar '26\mkern -9.75mu\lambda}}_{040}^{*}\right)}^\frac{1}{2}}$$ .
(iv) The difference type estimator given by:
  7 $${\mathrm{T}}_{3}=\left\{{{\raisebox{2.1pt}{$\upomega$}\kern-2.3pt\rotatebox{80}{\tiny $\smile$}}}_{1}{s}_{y}^{2}+{{\raisebox{2.1pt}{$\upomega$}\kern-2.3pt\rotatebox{80}{\tiny $\smile$}}}_{2}\left({S}_{x}^{2}-{s}_{x}^{2}\right)\right\},$$
  where $${{\raisebox{2.1pt}{$\upomega$}\kern-2.3pt\rotatebox{80}{\tiny $\smile$}}}_{1}$$ and $${{\raisebox{2.1pt}{$\upomega$}\kern-2.3pt\rotatebox{80}{\tiny $\smile$}}}_{2}$$ is the unidentified constants:
  $${{\raisebox{2.1pt}{$\upomega$}\kern-2.3pt\rotatebox{80}{\tiny $\smile$}}}_{1}=\frac{{{\mkern0.75mu\mathchar '26\mkern -9.75mu\lambda}}_{040}^{*}}{{\reflectbox{$\lambda$}}\left\{{{\mkern0.75mu\mathchar '26\mkern -9.75mu\lambda}}_{400}^{*}{{\mkern0.75mu\mathchar '26\mkern -9.75mu\lambda}}_{040}^{*}-{{\mkern0.75mu\mathchar '26\mkern -9.75mu\lambda}}_{220}^{*2}\right\}+{{\mkern0.75mu\mathchar '26\mkern -9.75mu\lambda}}_{040}^{*}},$$
  and
  $${{\raisebox{2.1pt}{$\upomega$}\kern-2.3pt\rotatebox{80}{\tiny $\smile$}}}_{2}=\frac{{S}_{y}^{2} {{\mkern0.75mu\mathchar '26\mkern -9.75mu\lambda}}_{220}^{*}}{{S}_{x}^{2}\left\{{\reflectbox{$\lambda$}}\left({{\mkern0.75mu\mathchar '26\mkern -9.75mu\lambda}}_{400}^{*}{{\mkern0.75mu\mathchar '26\mkern -9.75mu\lambda}}_{040}^{*}-{{\mkern0.75mu\mathchar '26\mkern -9.75mu\lambda}}_{220}^{*2}\right)+{{\mkern0.75mu\mathchar '26\mkern -9.75mu\lambda}}_{040}^{*}\right\}}.$$
  The bias and minimum MSE at the optimal value of $${{\raisebox{2.1pt}{$\upomega$}\kern-2.3pt\rotatebox{80}{\tiny $\smile$}}}_{1}$$ and $${{\raisebox{2.1pt}{$\upomega$}\kern-2.3pt\rotatebox{80}{\tiny $\smile$}}}_{2}$$, are given by:
  $$\mathrm{Bias}\left({\mathrm{T}}_{3}\right)={S}_{y}^{2}\left\{\frac{{{\mkern0.75mu\mathchar '26\mkern -9.75mu\lambda}}_{040}^{*}}{{\reflectbox{$\lambda$}}\left\{{{\mkern0.75mu\mathchar '26\mkern -9.75mu\lambda}}_{400}^{*}{{\mkern0.75mu\mathchar '26\mkern -9.75mu\lambda}}_{040}^{*}-{{\mkern0.75mu\mathchar '26\mkern -9.75mu\lambda}}_{220}^{*2}\right\}+{{\mkern0.75mu\mathchar '26\mkern -9.75mu\lambda}}_{040}^{*}}-1\right\},$$
  and
  8 $$\mathrm{MSE}\left({\mathrm{T}}_{3}\right)=\frac{{\reflectbox{$\lambda$}}{S}_{y}^{4} \left[{{\mkern0.75mu\mathchar '26\mkern -9.75mu\lambda}}_{400}^{*}{{\mkern0.75mu\mathchar '26\mkern -9.75mu\lambda}}_{040}^{*}-{{\mkern0.75mu\mathchar '26\mkern -9.75mu\lambda}}_{220}^{*2}\right]}{{\reflectbox{$\lambda$}}\left[{{\mkern0.75mu\mathchar '26\mkern -9.75mu\lambda}}_{400}^{*}{{\mkern0.75mu\mathchar '26\mkern -9.75mu\lambda}}_{040}^{*}-{{\mkern0.75mu\mathchar '26\mkern -9.75mu\lambda}}_{220}^{*2}\right]+{{\mkern0.75mu\mathchar '26\mkern -9.75mu\lambda}}_{040}^{*}}$$
(v) The exponential ratio and product type estimators by Singh et al.^24^:
  9 $${{\mathrm{T}}_{4}=s}_{y}^{2}\mathrm{exp}\left\{\frac{{S}_{x}^{2}-{s}_{x}^{2}}{{S}_{x}^{2}+{s}_{x}^{2}}\right\}$$
  10 $${{\mathrm{T}}_{5}=s}_{y}^{2}\mathrm{exp}\left\{\frac{{s}_{x}^{2}-{S}_{x}^{2}}{{s}_{x}^{2}+{S}_{x}^{2}}\right\}$$
  The bias and MSE of ${\mathrm{T}}_{4}$ and ${\mathrm{T}}_{5}$ are given by:
  11 $$\mathrm{Bias}\left({\mathrm{T}}_{4}\right)={\reflectbox{$\lambda$}}{S}_{y}^{2}\left\{\frac{3}{8}{{\mkern0.75mu\mathchar '26\mkern -9.75mu\lambda}}_{040}^{*}-\frac{1}{2}{{\mkern0.75mu\mathchar '26\mkern -9.75mu\lambda}}_{220}^{*}\right\},\mathrm{MSE}\left({\mathrm{T}}_{4}\right)={\reflectbox{$\lambda$}}{S}_{y}^{4}\left[{{\mkern0.75mu\mathchar '26\mkern -9.75mu\lambda}}_{400}^{*}+\frac{1}{4}{{\mkern0.75mu\mathchar '26\mkern -9.75mu\lambda}}_{040}^{*}-{{\mkern0.75mu\mathchar '26\mkern -9.75mu\lambda}}_{220}^{*}+\frac{1}{4}\right]$$
  and
  12 $$\mathrm{Bias}\left({\mathrm{T}}_{5}\right)={\reflectbox{$\lambda$}}{S}_{y}^{2}\left\{\frac{1}{2}{{\mkern0.75mu\mathchar '26\mkern -9.75mu\lambda}}_{220}^{*}-\frac{1}{8}{{\mkern0.75mu\mathchar '26\mkern -9.75mu\lambda}}_{040}^{*}\right\},\mathrm{MSE}\left({\mathrm{T}}_{5}\right)={\reflectbox{$\lambda$}}{S}_{y}^{4}\left[{{\mkern0.75mu\mathchar '26\mkern -9.75mu\lambda}}_{400}^{*}+\frac{1}{4}{{\mkern0.75mu\mathchar '26\mkern -9.75mu\lambda}}_{040}^{*}-{{\mkern0.75mu\mathchar '26\mkern -9.75mu\lambda}}_{220}^{*}+\frac{9}{4}\right]$$
(vi) Grover and Kaur^25^, suggested the following estimator, which is given by:
  13 $${\mathrm{T}}_{6}=\left[{{\raisebox{2.1pt}{$\upomega$}\kern-2.3pt\rotatebox{80}{\tiny $\smile$}}}_{3}{s}_{y}^{2}+{{\raisebox{2.1pt}{$\upomega$}\kern-2.3pt\rotatebox{80}{\tiny $\smile$}}}_{4}\left({S}_{x}^{2}-{s}_{x}^{2}\right)\right]\mathrm{exp}\left(\frac{{S}_{x}^{2}-{s}_{x}^{2}}{{S}_{x}^{2}+{s}_{x}^{2}}\right)$$
  The bias of ${\mathrm{T}}_{6}$, is given by:
  $$\mathrm{Bias}\left({\mathrm{T}}_{6}\right)=\left[-{S}_{y}^{2}+{{\raisebox{2.1pt}{$\upomega$}\kern-2.3pt\rotatebox{80}{\tiny $\smile$}}}_{3}{S}_{y}^{2}\left\{1+\frac{3}{8} {{\mkern0.75mu\mathchar '26\mkern -9.75mu\lambda}}_{040}^{*}-\frac{1}{2}{{\mkern0.75mu\mathchar '26\mkern -9.75mu\lambda}}_{220}^{*}\right\}+\frac{1}{2}{{\raisebox{2.1pt}{$\upomega$}\kern-2.3pt\rotatebox{80}{\tiny $\smile$}}}_{4}{S}_{x}^{2}{\reflectbox{$\lambda$}}{{\mkern0.75mu\mathchar '26\mkern -9.75mu\lambda}}_{040}^{*}\right],$$
  The minimum values of $${{\raisebox{2.1pt}{$\upomega$}\kern-2.3pt\rotatebox{80}{\tiny $\smile$}}}_{3}$$ and $${{\raisebox{2.1pt}{$\upomega$}\kern-2.3pt\rotatebox{80}{\tiny $\smile$}}}_{4}$$ are given by:
  $${{\raisebox{2.1pt}{$\upomega$}\kern-2.3pt\rotatebox{80}{\tiny $\smile$}}}_{3}=\frac{\left\{8{{\mkern0.75mu\mathchar '26\mkern -9.75mu\lambda}}_{040}^{*}-{\reflectbox{$\lambda$}}{{\mkern0.75mu\mathchar '26\mkern -9.75mu\lambda}}_{040}^{*2}\right\}}{8\left[{\reflectbox{$\lambda$}}\left({{\mkern0.75mu\mathchar '26\mkern -9.75mu\lambda}}_{400}^{*}{{\mkern0.75mu\mathchar '26\mkern -9.75mu\lambda}}_{040}^{*}-{{\mkern0.75mu\mathchar '26\mkern -9.75mu\lambda}}_{220}^{*2}\right)+{{\mkern0.75mu\mathchar '26\mkern -9.75mu\lambda}}_{040}^{*}\right]},$$
  and
  $${{\raisebox{2.1pt}{$\upomega$}\kern-2.3pt\rotatebox{80}{\tiny $\smile$}}}_{4}=\frac{{S}_{y}^{2}\left({\reflectbox{$\lambda$}}{{\mkern0.75mu\mathchar '26\mkern -9.75mu\lambda}}_{040}^{*2}-{\reflectbox{$\lambda$}}{{\mkern0.75mu\mathchar '26\mkern -9.75mu\lambda}}_{040}^{*}{{\mkern0.75mu\mathchar '26\mkern -9.75mu\lambda}}_{220}^{*}+8{{\mkern0.75mu\mathchar '26\mkern -9.75mu\lambda}}_{220}^{*}-4\left\{{{\mkern0.75mu\mathchar '26\mkern -9.75mu\lambda}}_{040}^{*}-{\reflectbox{$\lambda$}}\left({{\mkern0.75mu\mathchar '26\mkern -9.75mu\lambda}}_{400}^{*}{{\mkern0.75mu\mathchar '26\mkern -9.75mu\lambda}}_{040}^{*}-{{\mkern0.75mu\mathchar '26\mkern -9.75mu\lambda}}_{220}^{*2}\right)\right\}\right)}{8{ S}_{x}^{2}\left[{\reflectbox{$\lambda$}}\left\{{{\mkern0.75mu\mathchar '26\mkern -9.75mu\lambda}}_{400}^{*}{{\mkern0.75mu\mathchar '26\mkern -9.75mu\lambda}}_{040}^{*}-{{\mkern0.75mu\mathchar '26\mkern -9.75mu\lambda}}_{220}^{*2}\right\}+{{\mkern0.75mu\mathchar '26\mkern -9.75mu\lambda}}_{040}^{*}\right]}.$$
  The minimum MSE at the optimum values of $${{\raisebox{2.1pt}{$\upomega$}\kern-2.3pt\rotatebox{80}{\tiny $\smile$}}}_{3}$$ and $${{\raisebox{2.1pt}{$\upomega$}\kern-2.3pt\rotatebox{80}{\tiny $\smile$}}}_{4}$$, are given by:
  14 $$\mathrm{MSE}\left({\mathrm{T}}_{6}\right)=\frac{{S}_{y}^{4}\left[64\left\{{{\mkern0.75mu\mathchar '26\mkern -9.75mu\lambda}}_{400}^{*}{{\mkern0.75mu\mathchar '26\mkern -9.75mu\lambda}}_{040}^{*}-{{\mkern0.75mu\mathchar '26\mkern -9.75mu\lambda}}_{220}^{*2}\right\}-{\reflectbox{$\lambda$}}{{\mkern0.75mu\mathchar '26\mkern -9.75mu\lambda}}_{040}^{*3}-16{\reflectbox{$\lambda$}}{{\mkern0.75mu\mathchar '26\mkern -9.75mu\lambda}}_{040}^{*} \left\{{{\mkern0.75mu\mathchar '26\mkern -9.75mu\lambda}}_{400}^{*}{{\mkern0.75mu\mathchar '26\mkern -9.75mu\lambda}}_{040}^{*}-{{\mkern0.75mu\mathchar '26\mkern -9.75mu\lambda}}_{220}^{*2}\right\}\right]}{64\left\{{\reflectbox{$\lambda$}}\left({{\mkern0.75mu\mathchar '26\mkern -9.75mu\lambda}}_{400}^{*}{{\mkern0.75mu\mathchar '26\mkern -9.75mu\lambda}}_{040}^{*}-{{\mkern0.75mu\mathchar '26\mkern -9.75mu\lambda}}_{220}^{*2}\right)+{{\mkern0.75mu\mathchar '26\mkern -9.75mu\lambda}}_{040}^{*}\right\}}.$$

## Proposed estimator

The use of auxiliary variables can improve the precision and efficiency of an estimator during both the design and estimation procedures. Taking motivation from the work of Ahmad et al.^26^, we propose an enhanced estimator for estimation of finite population variance using two auxiliary variables under simple random sampling. Our suggested variance estimator based on simple random sampling is more efficient and flexible as compared to all the existing estimators considered in this study, which is given by:

15 $${\mathrm{T}}_{prop }=\left[{{\raisebox{2.1pt}{$\upomega$}\kern-2.3pt\rotatebox{80}{\tiny $\smile$}}}_{5}{s}_{y}^{2}+{{\raisebox{2.1pt}{$\upomega$}\kern-2.3pt\rotatebox{80}{\tiny $\smile$}}}_{6}\left({S}_{x1}^{2}-{s}_{x1}^{2}\right)+{{\raisebox{2.1pt}{$\upomega$}\kern-2.3pt\rotatebox{80}{\tiny $\smile$}}}_{7}\left({S}_{x2}^{2}-{s}_{x2}^{2}\right)\right]\mathrm{exp}\left(\frac{{S}_{x1}^{2}-{s}_{x1}^{2}}{{S}_{x1}^{2}+{s}_{x1}^{2}}\right),$$

where $${{\raisebox{2.1pt}{$\upomega$}\kern-2.3pt\rotatebox{80}{\tiny $\smile$}}}_{5}$$, $${{\raisebox{2.1pt}{$\upomega$}\kern-2.3pt\rotatebox{80}{\tiny $\smile$}}}_{6}$$ and $${{\raisebox{2.1pt}{$\upomega$}\kern-2.3pt\rotatebox{80}{\tiny $\smile$}}}_{7}$$ are unknown constants.

$${\mathrm{T}}_{prop}=\left[{{\raisebox{2.1pt}{$\upomega$}\kern-2.3pt\rotatebox{80}{\tiny $\smile$}}}_{5}{S}_{y}^{2}\left(1+{\xi }_{o}\right)-{{\raisebox{2.1pt}{$\upomega$}\kern-2.3pt\rotatebox{80}{\tiny $\smile$}}}_{6}{{S}_{x1}^{2}\xi }_{1}-{{\raisebox{2.1pt}{$\upomega$}\kern-2.3pt\rotatebox{80}{\tiny $\smile$}}}_{7}{S}_{x1}^{2} {\xi }_{2}\right]\left(1-\frac{{\xi }_{1} }{2}+\frac{3{\xi }_{1}^{2}}{8}+\dots \right).$$

After expanding the above equation, we have

16 $$\begin{aligned}\left({\mathrm{T}}_{prop}-{S}_{y}^{2}\right) & =-{S}_{y}^{2}+{{\raisebox{2.1pt}{$\upomega$}\kern-2.3pt\rotatebox{80}{\tiny $\smile$}}}_{5}{S}_{y}^{2}+{{\raisebox{2.1pt}{$\upomega$}\kern-2.3pt\rotatebox{80}{\tiny $\smile$}}}_{5}{S}_{y}^{2}{\xi }_{o}-\frac{1}{2}{{\raisebox{2.1pt}{$\upomega$}\kern-2.3pt\rotatebox{80}{\tiny $\smile$}}}_{5}{S}_{y}^{2}{\xi }_{1}-{{\raisebox{2.1pt}{$\upomega$}\kern-2.3pt\rotatebox{80}{\tiny $\smile$}}}_{6}{{S}_{x1}^{2}\xi }_{1}-{ {\raisebox{2.1pt}{$\upomega$}\kern-2.3pt\rotatebox{80}{\tiny $\smile$}}}_{7}{{S}_{x2}^{2}\xi }_{2}+\frac{1}{2}{{\raisebox{2.1pt}{$\upomega$}\kern-2.3pt\rotatebox{80}{\tiny $\smile$}}}_{5}{S}_{y}^{2} {\xi }_{o}{\xi }_{1}\\ &\quad+\frac{3}{8}{{\raisebox{2.1pt}{$\upomega$}\kern-2.3pt\rotatebox{80}{\tiny $\smile$}}}_{5}{S}_{y}^{2} {\xi }_{1}^{2}+\frac{1}{2}{{\raisebox{2.1pt}{$\upomega$}\kern-2.3pt\rotatebox{80}{\tiny $\smile$}}}_{6}{S}_{x1}^{2}{\xi }_{1}^{2}+\frac{1}{2}{{\raisebox{2.1pt}{$\upomega$}\kern-2.3pt\rotatebox{80}{\tiny $\smile$}}}_{7}{S}_{x2}^{2}{\xi }_{1}{\xi }_{2}\end{aligned}$$

The bias and mean square error of ${\mathrm{T}}_{prop}$, is given by:

17 $$\begin{aligned}\mathrm{Bias}\left({\mathrm{T}}_{prop}\right)&\cong {S}_{y}^{2}\left({{\raisebox{2.1pt}{$\upomega$}\kern-2.3pt\rotatebox{80}{\tiny $\smile$}}}_{5}-1\right)+\frac{3}{8}{S}_{y}^{2}{{\raisebox{2.1pt}{$\upomega$}\kern-2.3pt\rotatebox{80}{\tiny $\smile$}}}_{5}{\reflectbox{$\lambda$}}{ {\mkern0.75mu\mathchar '26\mkern -9.75mu\lambda}}_{040}^{*}+\frac{1}{2}{S}_{x}^{2}{{\raisebox{2.1pt}{$\upomega$}\kern-2.3pt\rotatebox{80}{\tiny $\smile$}}}_{6}{\reflectbox{$\lambda$}}{{\mkern0.75mu\mathchar '26\mkern -9.75mu\lambda}}_{040}^{*}-{\frac{1}{2}S}_{y}^{2}{{\raisebox{2.1pt}{$\upomega$}\kern-2.3pt\rotatebox{80}{\tiny $\smile$}}}_{5}{\reflectbox{$\lambda$}}{{\mkern0.75mu\mathchar '26\mkern -9.75mu\lambda}}_{220}^{*}+\frac{1}{2}{S}_{x2}^{2}{\reflectbox{$\lambda$}}{{\mkern0.75mu\mathchar '26\mkern -9.75mu\lambda}}_{022}^{*}\nonumber\\ \mathrm{MSE}\left({\mathrm{T}}_{prop}\right)&\cong {S}_{y}^{4}{\left({{\raisebox{2.1pt}{$\upomega$}\kern-2.3pt\rotatebox{80}{\tiny $\smile$}}}_{5}-1\right)}^{2}+{S}_{y}^{4}{\reflectbox{$\lambda$}}{ {\mkern0.75mu\mathchar '26\mkern -9.75mu\lambda}}_{400}^{*}{{\raisebox{2.1pt}{$\upomega$}\kern-2.3pt\rotatebox{80}{\tiny $\smile$}}}_{5}^{2}+{S}_{x1}^{4}{\reflectbox{$\lambda$}}{ {\mkern0.75mu\mathchar '26\mkern -9.75mu\lambda}}_{040}^{*}{{\raisebox{2.1pt}{$\upomega$}\kern-2.3pt\rotatebox{80}{\tiny $\smile$}}}_{6}+{S}_{x2}^{2}{\reflectbox{$\lambda$}}{ {\mkern0.75mu\mathchar '26\mkern -9.75mu\lambda}}_{004}^{*}{{\raisebox{2.1pt}{$\upomega$}\kern-2.3pt\rotatebox{80}{\tiny $\smile$}}}_{7} +{S}_{y}^{4}{{\raisebox{2.1pt}{$\upomega$}\kern-2.3pt\rotatebox{80}{\tiny $\smile$}}}_{5}^{2}{\reflectbox{$\lambda$}}{ {\mkern0.75mu\mathchar '26\mkern -9.75mu\lambda}}_{040}^{*}-{S}_{y}^{2}{S}_{x1}^{2}{{\raisebox{2.1pt}{$\upomega$}\kern-2.3pt\rotatebox{80}{\tiny $\smile$}}}_{6}{\reflectbox{$\lambda$}}{ {\mkern0.75mu\mathchar '26\mkern -9.75mu\lambda}}_{040}^{*}\nonumber\\&\quad+ 2{S}_{y}^{2}{S}_{x1}^{2}{{\raisebox{2.1pt}{$\upomega$}\kern-2.3pt\rotatebox{80}{\tiny $\smile$}}}_{5}{{\raisebox{2.1pt}{$\upomega$}\kern-2.3pt\rotatebox{80}{\tiny $\smile$}}}_{6}{\reflectbox{$\lambda$}}{ {\mkern0.75mu\mathchar '26\mkern -9.75mu\lambda}}_{040}^{*} -\frac{3}{4}{S}_{y}^{4}{{\raisebox{2.1pt}{$\upomega$}\kern-2.3pt\rotatebox{80}{\tiny $\smile$}}}_{5}{\reflectbox{$\lambda$}}{ {\mkern0.75mu\mathchar '26\mkern -9.75mu\lambda}}_{040}^{*}+{S}_{y}^{4}{{\raisebox{2.1pt}{$\upomega$}\kern-2.3pt\rotatebox{80}{\tiny $\smile$}}}_{5}{\reflectbox{$\lambda$}}{ {\mkern0.75mu\mathchar '26\mkern -9.75mu\lambda}}_{220}^{*}-2{S}_{y}^{4}{{\raisebox{2.1pt}{$\upomega$}\kern-2.3pt\rotatebox{80}{\tiny $\smile$}}}_{5}^{2} 2{\reflectbox{$\lambda$}}{ {\mkern0.75mu\mathchar '26\mkern -9.75mu\lambda}}_{220}^{*}-2{S}_{y}^{2}{S}_{x1}^{2}{{\raisebox{2.1pt}{$\upomega$}\kern-2.3pt\rotatebox{80}{\tiny $\smile$}}}_{5}{{\raisebox{2.1pt}{$\upomega$}\kern-2.3pt\rotatebox{80}{\tiny $\smile$}}}_{6}{\reflectbox{$\lambda$}}{ {\mkern0.75mu\mathchar '26\mkern -9.75mu\lambda}}_{220}^{*}\nonumber\\&\quad-2{S}_{y}^{2}{S}_{x2}^{2}{{\raisebox{2.1pt}{$\upomega$}\kern-2.3pt\rotatebox{80}{\tiny $\smile$}}}_{5}{{\raisebox{2.1pt}{$\upomega$}\kern-2.3pt\rotatebox{80}{\tiny $\smile$}}}_{7}{\reflectbox{$\lambda$}}{ {\mkern0.75mu\mathchar '26\mkern -9.75mu\lambda}}_{202}^{*}-{S}_{y}^{2}{S}_{x2}^{2}{{\raisebox{2.1pt}{$\upomega$}\kern-2.3pt\rotatebox{80}{\tiny $\smile$}}}_{7}{\reflectbox{$\lambda$}}{ {\mkern0.75mu\mathchar '26\mkern -9.75mu\lambda}}_{022}^{*}+2{S}_{y}^{2}{S}_{x2}^{2}{{\raisebox{2.1pt}{$\upomega$}\kern-2.3pt\rotatebox{80}{\tiny $\smile$}}}_{5}{{\raisebox{2.1pt}{$\upomega$}\kern-2.3pt\rotatebox{80}{\tiny $\smile$}}}_{7}{\reflectbox{$\lambda$}}{ {\mkern0.75mu\mathchar '26\mkern -9.75mu\lambda}}_{022}^{*}-2{S}_{y}^{2}{S}_{x2}^{2}{{\raisebox{2.1pt}{$\upomega$}\kern-2.3pt\rotatebox{80}{\tiny $\smile$}}}_{6}{{\raisebox{2.1pt}{$\upomega$}\kern-2.3pt\rotatebox{80}{\tiny $\smile$}}}_{7}{\reflectbox{$\lambda$}}{ {\mkern0.75mu\mathchar '26\mkern -9.75mu\lambda}}_{022}^{*}\end{aligned}$$

The optimal values of $${{\raisebox{2.1pt}{$\upomega$}\kern-2.3pt\rotatebox{80}{\tiny $\smile$}}}_{5}$$, $${{\raisebox{2.1pt}{$\upomega$}\kern-2.3pt\rotatebox{80}{\tiny $\smile$}}}_{6}$$, and $${{\raisebox{2.1pt}{$\upomega$}\kern-2.3pt\rotatebox{80}{\tiny $\smile$}}}_{7}$$ are obtained by minimizing (17), and are given by:

$${{{\raisebox{2.1pt}{$\upomega$}\kern-2.3pt\rotatebox{80}{\tiny $\smile$}}}_{5}}_{(Opt)}=\frac{8-{\reflectbox{$\lambda$}}{{\mkern0.75mu\mathchar '26\mkern -9.75mu\lambda}}_{040}^{*}}{{{\mkern0.75mu\mathchar '26\mkern -9.75mu\lambda}}_{400 }^{*}\left[8\left\{\frac{1}{{{\mkern0.75mu\mathchar '26\mkern -9.75mu\lambda}}_{400}^{*}}+{\reflectbox{$\lambda$}}\left({\upgamma }^{*} +1\right)\right\}\right]},$$

$${{{\raisebox{2.1pt}{$\upomega$}\kern-2.3pt\rotatebox{80}{\tiny $\smile$}}}_{6}}_{(Opt)}=\frac{{S}_{y}^{2}\left[\begin{array}{c}{\reflectbox{$\lambda$}} {{\mkern0.75mu\mathchar '26\mkern -9.75mu\lambda}}_{040}^{*}\left({{\mkern0.75mu\mathchar '26\mkern -9.75mu\lambda}}_{040}^{*}{{\mkern0.75mu\mathchar '26\mkern -9.75mu\lambda}}_{004}^{*}-{{\mkern0.75mu\mathchar '26\mkern -9.75mu\lambda}}_{220}^{*2}\right)+\left({{\mkern0.75mu\mathchar '26\mkern -9.75mu\lambda}}_{004}^{*}{{\mkern0.75mu\mathchar '26\mkern -9.75mu\lambda}}_{220}^{*}-{{\mkern0.75mu\mathchar '26\mkern -9.75mu\lambda}}_{202}^{*}{{\mkern0.75mu\mathchar '26\mkern -9.75mu\lambda}}_{022}^{*}\right)\left(8-{\reflectbox{$\lambda$}}{ {\mkern0.75mu\mathchar '26\mkern -9.75mu\lambda}}_{040}^{*}\right)+4{{\mkern0.75mu\mathchar '26\mkern -9.75mu\lambda}}_{400}^{*}\left({{\mkern0.75mu\mathchar '26\mkern -9.75mu\lambda}}_{040}^{*}{{\mkern0.75mu\mathchar '26\mkern -9.75mu\lambda}}_{004-}^{*}{{\mkern0.75mu\mathchar '26\mkern -9.75mu\lambda}}_{022}^{*2}\right)\\ \left\{\left(\frac{-1}{{{\mkern0.75mu\mathchar '26\mkern -9.75mu\lambda}}_{400}^{*}}\right)+{\reflectbox{$\lambda$}}\left({\upgamma }^{*} +1\right) \right\}\end{array}\right]}{8{S}_{x}^{2}{{\mkern0.75mu\mathchar '26\mkern -9.75mu\lambda}}_{400}^{*}\left({{\mkern0.75mu\mathchar '26\mkern -9.75mu\lambda}}_{040}^{*}{{\mkern0.75mu\mathchar '26\mkern -9.75mu\lambda}}_{004}^{*}-{{\mkern0.75mu\mathchar '26\mkern -9.75mu\lambda}}_{220}^{*2}\right)\left\{\left(\frac{1}{{{\mkern0.75mu\mathchar '26\mkern -9.75mu\lambda}}_{400}^{*}}\right)+{\reflectbox{$\lambda$}}\left({\upgamma }^{*} +1\right) \right\}},$$

and

$${{{\raisebox{2.1pt}{$\upomega$}\kern-2.3pt\rotatebox{80}{\tiny $\smile$}}}_{7}}_{(Opt)}=\frac{{S}_{y}^{2}\left[8-{\reflectbox{$\lambda$}}{{\mkern0.75mu\mathchar '26\mkern -9.75mu\lambda}}_{040}^{*}\right]\left\{{{\mkern0.75mu\mathchar '26\mkern -9.75mu\lambda}}_{220}^{*}{{\mkern0.75mu\mathchar '26\mkern -9.75mu\lambda}}_{002}^{*}-{{\mkern0.75mu\mathchar '26\mkern -9.75mu\lambda}}_{040}^{*}{{\mkern0.75mu\mathchar '26\mkern -9.75mu\lambda}}_{202}^{*}\right\}}{8{S}_{rx}^{2}{{\mkern0.75mu\mathchar '26\mkern -9.75mu\lambda}}_{400}^{*}\left({{\mkern0.75mu\mathchar '26\mkern -9.75mu\lambda}}_{040}^{*}{{\mkern0.75mu\mathchar '26\mkern -9.75mu\lambda}}_{004}^{*}-{{\mkern0.75mu\mathchar '26\mkern -9.75mu\lambda}}_{220}^{*2}\right)\left\{\left(\frac{1}{{{\mkern0.75mu\mathchar '26\mkern -9.75mu\lambda}}_{400}^{*}}\right)+{\reflectbox{$\lambda$}}\left({\upgamma }^{*} +1\right) \right\}}.$$

The minimal MSE at the optimum values of $${{{\raisebox{2.1pt}{$\upomega$}\kern-2.3pt\rotatebox{80}{\tiny $\smile$}}}_{5}}_{(Opt)}$$, $${{{\raisebox{2.1pt}{$\upomega$}\kern-2.3pt\rotatebox{80}{\tiny $\smile$}}}_{6}}_{(Opt)}$$ and $${{{\raisebox{2.1pt}{$\upomega$}\kern-2.3pt\rotatebox{80}{\tiny $\smile$}}}_{7}}_{(Opt)}$$, are given by:

18 $$\mathrm{MSE}\left({\mathrm{T}}_{prop}\right)=\frac{{\reflectbox{$\lambda$}}{S}_{y}^{4}\left[64\left({\upgamma }^{*}+1\right)-{\reflectbox{$\lambda$}}\left(\frac{{{\mkern0.75mu\mathchar '26\mkern -9.75mu\lambda}}_{040}^{*2}}{{{\mkern0.75mu\mathchar '26\mkern -9.75mu\lambda}}_{400}^{*}}\right)-16 {\reflectbox{$\lambda$}}{{\mkern0.75mu\mathchar '26\mkern -9.75mu\lambda}}_{040}^{*}\left({\upgamma }^{*}+1\right)\right]}{64\left\{\frac{1}{{{\mkern0.75mu\mathchar '26\mkern -9.75mu\lambda}}_{400}^{*}}+{\reflectbox{$\lambda$}}\left({\upgamma }^{*}+1\right)\right\}},$$

where

$${\upgamma }^{*}=\frac{2{{\mkern0.75mu\mathchar '26\mkern -9.75mu\lambda}}_{220}^{*}{{\mkern0.75mu\mathchar '26\mkern -9.75mu\lambda}}_{202}^{*}{{\mkern0.75mu\mathchar '26\mkern -9.75mu\lambda}}_{022}^{*}-{{\mkern0.75mu\mathchar '26\mkern -9.75mu\lambda}}_{040}^{*}{{\mkern0.75mu\mathchar '26\mkern -9.75mu\lambda}}_{202}^{*2}-{{\mkern0.75mu\mathchar '26\mkern -9.75mu\lambda}}_{004}^{*}{{\mkern0.75mu\mathchar '26\mkern -9.75mu\lambda}}_{220}^{*2}}{{{\mkern0.75mu\mathchar '26\mkern -9.75mu\lambda}}_{400}^{*}\left({{\mkern0.75mu\mathchar '26\mkern -9.75mu\lambda}}_{040}^{*}{{\mkern0.75mu\mathchar '26\mkern -9.75mu\lambda}}_{004 -}^{*}{{\mkern0.75mu\mathchar '26\mkern -9.75mu\lambda}}_{220}^{*2}\right)}.$$

## Efficiency assessment

In this section, we compare the proposed estimator with existing estimators in terms of mean square error.

1. By taking (2) and (18)
  $$\mathrm{Var}\left({\mathrm{T}}_{0}\right)-\mathrm{MSE}\left({\mathrm{T}}_{prop}\right)>0$$
  19 $${S}_{y}^{4}{\reflectbox{$\lambda$}}{{\mkern0.75mu\mathchar '26\mkern -9.75mu\lambda}}_{400}^{*}-\frac{{\reflectbox{$\lambda$}}{S}_{y}^{2}\left[64\left({\upgamma }^{*}+1\right)-{\reflectbox{$\lambda$}}\left(\frac{{{\mkern0.75mu\mathchar '26\mkern -9.75mu\lambda}}_{040}^{*2}}{{{\mkern0.75mu\mathchar '26\mkern -9.75mu\lambda}}_{400}^{*}}\right)-16 {\reflectbox{$\lambda$}}{{\mkern0.75mu\mathchar '26\mkern -9.75mu\lambda}}_{040}^{*}\left({\upgamma }^{*}+1\right)\right]}{64\left\{\frac{1}{{{\mkern0.75mu\mathchar '26\mkern -9.75mu\lambda}}_{400}^{*}}+{\reflectbox{$\lambda$}}\left({\upgamma }^{*}+1\right)\right\}}>0$$
  20 $$\frac{{\reflectbox{$\lambda$}}{S}_{y}^{2}\left[64{S}_{y}^{2}{{\mkern0.75mu\mathchar '26\mkern -9.75mu\lambda}}_{400}^{*}\left\{{\Psi }_{1}\right\}+\left[16{\reflectbox{$\lambda$}}{{\mkern0.75mu\mathchar '26\mkern -9.75mu\lambda}}_{040}^{*}\left({\upgamma }^{*}+1\right)-64{\upgamma }^{*}+{\reflectbox{$\lambda$}}\left(\frac{{{\reflectbox{$\lambda$}}}_{040}^{*2}}{{{\mkern0.75mu\mathchar '26\mkern -9.75mu\lambda}}_{400}^{*}}\right)-64\right]\right]}{64{\Psi }_{1}}>0$$
  where $${\Psi }_{1}$$=$$\frac{1}{{{\mkern0.75mu\mathchar '26\mkern -9.75mu\lambda}}_{400}^{*}}+{\reflectbox{$\lambda$}}\left({\upgamma }^{*}+1\right),$$
  $${\Psi }_{2}=\left[64{\upgamma }^{*}+64-{\reflectbox{$\lambda$}}\left(\frac{{{\mkern0.75mu\mathchar '26\mkern -9.75mu\lambda}}_{040}^{*2}}{{{\mkern0.75mu\mathchar '26\mkern -9.75mu\lambda}}_{400}^{*}}\right)-16{\reflectbox{$\lambda$}}{{\mkern0.75mu\mathchar '26\mkern -9.75mu\lambda}}_{040}^{*}\left({\upgamma }^{*}+1\right)\right],$$
  $${\Psi }_{3}=\frac{1}{4}{\reflectbox{$\lambda$}}{{\mkern0.75mu\mathchar '26\mkern -9.75mu\lambda}}_{040}^{*}\left({\upgamma }^{*}+1\right)-{\upgamma }^{*}+\frac{1}{64}{\reflectbox{$\lambda$}}\left(\frac{{{\mkern0.75mu\mathchar '26\mkern -9.75mu\lambda}}_{040}^{*2}}{{{\mkern0.75mu\mathchar '26\mkern -9.75mu\lambda}}_{400}^{*}}\right)-1$$
2. By taking (4) and (18)
  $$\mathrm{MSE}\left({\mathrm{T}}_{1}\right)-\mathrm{MSE}\left({\mathrm{T}}_{prop}\right)>0$$
  21 $${\reflectbox{$\lambda$}}{S}_{y}^{4} \left\{{{\mkern0.75mu\mathchar '26\mkern -9.75mu\lambda}}_{400}^{*}+{{\mkern0.75mu\mathchar '26\mkern -9.75mu\lambda}}_{040}^{*}-2{{\mkern0.75mu\mathchar '26\mkern -9.75mu\lambda}}_{220}^{*}\right\}-\frac{{\reflectbox{$\lambda$}}{S}_{y}^{2}\left[64\left({\upgamma }^{*}+1\right)-{\reflectbox{$\lambda$}}\left(\frac{{{\mkern0.75mu\mathchar '26\mkern -9.75mu\lambda}}_{040}^{*2}}{{{\mkern0.75mu\mathchar '26\mkern -9.75mu\lambda}}_{400}^{*}}\right)-16 {\reflectbox{$\lambda$}}{{\mkern0.75mu\mathchar '26\mkern -9.75mu\lambda}}_{040}^{*}\left({\upgamma }^{*}+1\right)\right]}{64{\Psi }_{1}}>0$$
  22 $$\frac{{\reflectbox{$\lambda$}}{S}_{y}^{2}\left({S}_{y}^{2}\left\{{\mathcal{Y}}\right\}{\Psi }_{1}+{\Psi }_{3}\right)}{{\Psi }_{1}}>0$$
3. By taking (6) and (18)
  $$\mathrm{Var}\left({\mathrm{T}}_{2}\right)-\mathrm{MSE}\left({\mathrm{T}}_{prop}\right)>0$$
  23 $${S}_{y}^{4}{{\reflectbox{$\lambda$}}{\mkern0.75mu\mathchar '26\mkern -9.75mu\lambda}}_{400}^{*}\left(1-{\rho }^{2}\right)-\frac{{\reflectbox{$\lambda$}}{S}_{y}^{2}\left[64\left({\upgamma }^{*}+1\right)-{\reflectbox{$\lambda$}}\left(\frac{{{\mkern0.75mu\mathchar '26\mkern -9.75mu\lambda}}_{040}^{*2}}{{{\mkern0.75mu\mathchar '26\mkern -9.75mu\lambda}}_{400}^{*}}\right)-16 {\reflectbox{$\lambda$}}{{\mkern0.75mu\mathchar '26\mkern -9.75mu\lambda}}_{040}^{*}\left({\upgamma }^{*}+1\right)\right]}{64\left\{\frac{1}{{{\mkern0.75mu\mathchar '26\mkern -9.75mu\lambda}}_{400}^{*}}+{\reflectbox{$\lambda$}}\left({\upgamma }^{*}+1\right)\right\}}>0$$
  24 $$\frac{{\reflectbox{$\lambda$}}{S}_{y}^{2}\left({S}_{y}^{2}{{\mkern0.75mu\mathchar '26\mkern -9.75mu\lambda}}_{400}^{*}\left(1-{\rho }^{2}\right){\Psi }_{1}+{\Psi }_{3}\right)}{{\Psi }_{1}}>0$$
4. By taking (8) and (18)
  $$\mathrm{MSE}\left({\mathrm{T}}_{3}\right)-\mathrm{MSE}\left({\mathrm{T}}_{prop}\right)>0$$
  25 $$\frac{{\reflectbox{$\lambda$}}{S}_{y}^{4}\left[{\mathcal{Y}}\right]}{{\reflectbox{$\lambda$}}\left[{\mathcal{Y}}\right]+{{\mkern0.75mu\mathchar '26\mkern -9.75mu\lambda}}_{040}^{*}}-\frac{{\reflectbox{$\lambda$}}{S}_{y}^{2}\left[64\left({\upgamma }^{*}+1\right)-{\reflectbox{$\lambda$}}\left(\frac{{{\mkern0.75mu\mathchar '26\mkern -9.75mu\lambda}}_{040}^{*2}}{{{\mkern0.75mu\mathchar '26\mkern -9.75mu\lambda}}_{400}^{*}}\right)-16 {\reflectbox{$\lambda$}}{{\mkern0.75mu\mathchar '26\mkern -9.75mu\lambda}}_{040}^{*}\left({\upgamma }^{*}+1\right)\right]}{64\left\{\frac{1}{{{\mkern0.75mu\mathchar '26\mkern -9.75mu\lambda}}_{400}^{*}}+{\reflectbox{$\lambda$}}\left({\upgamma }^{*}+1\right)\right\}}>0$$
  26 $$\frac{\frac{1}{64}\left[{\reflectbox{$\lambda$}}{S}_{y}^{2}\left({{\mkern0.75mu\mathchar '26\mkern -9.75mu\lambda}}_{040}^{*}{\Psi }_{2}\right)+{{\Psi }_{1}S}_{y}^{2}+{\Psi }_{2}\left({\mathcal{Y}}\right)\right]}{{\reflectbox{$\lambda$}}(\left[{\mathcal{Y}}\right]+{{\mkern0.75mu\mathchar '26\mkern -9.75mu\lambda}}_{040}^{*}){\Psi }_{1}}>0.$$
  where $$\mathcal{Y}$$ = $${{\mkern0.75mu\mathchar '26\mkern -9.75mu\lambda}}_{400}^{*}{{\mkern0.75mu\mathchar '26\mkern -9.75mu\lambda}}_{040}^{*}-{{\mkern0.75mu\mathchar '26\mkern -9.75mu\lambda}}_{220}^{*2}$$.
5. By taking (11) and (18)
  $$\mathrm{MSE}\left({\mathrm{T}}_{4}\right)-\mathrm{MSE}\left({\mathrm{T}}_{prop}\right)>0$$
  27 $${\reflectbox{$\lambda$}}{S}_{y}^{4}\left[{{\mkern0.75mu\mathchar '26\mkern -9.75mu\lambda}}_{400}^{*}+\frac{1}{4}{{\mkern0.75mu\mathchar '26\mkern -9.75mu\lambda}}_{040}^{*}-{{\mkern0.75mu\mathchar '26\mkern -9.75mu\lambda}}_{220}^{*}+\frac{1}{4} \right]-\frac{{\reflectbox{$\lambda$}}{S}_{y}^{2}\left[64\left({\upgamma }^{*}+1\right)-{\reflectbox{$\lambda$}}\left(\frac{{{\mkern0.75mu\mathchar '26\mkern -9.75mu\lambda}}_{040}^{*2}}{{{\mkern0.75mu\mathchar '26\mkern -9.75mu\lambda}}_{400}^{*}}\right)-16 {\reflectbox{$\lambda$}}{{\mkern0.75mu\mathchar '26\mkern -9.75mu\lambda}}_{040}^{*}\left({\upgamma }^{*}+1\right)\right]}{64\left\{\frac{1}{{{\mkern0.75mu\mathchar '26\mkern -9.75mu\lambda}}_{400}^{*}}+ {\reflectbox{$\lambda$}}\left({\upgamma }^{*}+1\right)\right\}}>0$$
  28 $$\frac{{\reflectbox{$\lambda$}}{S}_{y}^{2}\left[{S}_{y}^{2}\left\{{{\mkern0.75mu\mathchar '26\mkern -9.75mu\lambda}}_{400}^{*}+\frac{1}{4}{{\mkern0.75mu\mathchar '26\mkern -9.75mu\lambda}}_{040}^{*}-{{\mkern0.75mu\mathchar '26\mkern -9.75mu\lambda}}_{220}^{*}+\frac{1}{ 4} \right\}{\Psi }_{1}+{\Psi }_{3} \right]}{{\Psi }_{1}}>0$$
6. By taking (12) and (18)
  $$\mathrm{MSE}\left({\mathrm{T}}_{5}\right)-\mathrm{MSE}\left({\mathrm{T}}_{prop}\right)>0$$
  29 $${\reflectbox{$\lambda$}}{S}_{y}^{4}\left[{{\mkern0.75mu\mathchar '26\mkern -9.75mu\lambda}}_{400}^{*}+\frac{1}{4}{{\mkern0.75mu\mathchar '26\mkern -9.75mu\lambda}}_{040}^{*}-{{\mkern0.75mu\mathchar '26\mkern -9.75mu\lambda}}_{220}^{*}+\frac{9}{4} \right]-\frac{{\reflectbox{$\lambda$}}{S}_{y}^{2}\left[64\left({\upgamma }^{*}+1\right)-{\reflectbox{$\lambda$}}\left(\frac{{{\mkern0.75mu\mathchar '26\mkern -9.75mu\lambda}}_{040h}^{*2}}{{{\mkern0.75mu\mathchar '26\mkern -9.75mu\lambda}}_{400h}^{*}}\right)-16 {\reflectbox{$\lambda$}}{{\mkern0.75mu\mathchar '26\mkern -9.75mu\lambda}}_{040h}^{*}\left({\upgamma }^{*}+1\right)\right]}{64\left\{\frac{1}{{{\mkern0.75mu\mathchar '26\mkern -9.75mu\lambda}}_{400h}^{*}}+{\reflectbox{$\lambda$}}\left({\upgamma }^{*}+1\right)\right\}}>0$$
  30 $$\frac{{\reflectbox{$\lambda$}}{S}_{y}^{2}\left[{S}_{y}^{2}\left\{{{\mkern0.75mu\mathchar '26\mkern -9.75mu\lambda}}_{400}^{*}+\frac{1}{4}{{\mkern0.75mu\mathchar '26\mkern -9.75mu\lambda}}_{040}^{*}-{{\mkern0.75mu\mathchar '26\mkern -9.75mu\lambda}}_{220}^{*}+\frac{9}{4} \right\}{\Psi }_{1}+{\Psi }_{3} \right]}{{\Psi }_{1}}>0$$
7. By taking (14) and (18)
  $$\mathrm{MSE}\left({\mathrm{T}}_{6}\right)-\mathrm{MSE}\left({\mathrm{T}}_{prop}\right)>0$$
  31 $$\frac{{S}_{y}^{4}\left[64\left\{{\mathcal{Y}}\right\}-{\reflectbox{$\lambda$}}{{\mkern0.75mu\mathchar '26\mkern -9.75mu\lambda}}_{040}^{*3}-16 {\reflectbox{$\lambda$}}{{\mkern0.75mu\mathchar '26\mkern -9.75mu\lambda}}_{040}^{*} \left\{{\mathcal{Y}}\right\}\right]}{64\left\{{\reflectbox{$\lambda$}}\left({\mathcal{Y}}\right)+{{\mkern0.75mu\mathchar '26\mkern -9.75mu\lambda}}_{040}^{*}\right\}}-\frac{{\reflectbox{$\lambda$}}{S}_{y}^{4}\left[64\left({\upgamma }^{*}+1\right)-{\reflectbox{$\lambda$}}\left(\frac{{{\mkern0.75mu\mathchar '26\mkern -9.75mu\lambda}}_{040}^{*2}}{{{\mkern0.75mu\mathchar '26\mkern -9.75mu\lambda}}_{400}^{*}}\right)-16 {\reflectbox{$\lambda$}}{{\mkern0.75mu\mathchar '26\mkern -9.75mu\lambda}}_{040}^{*}\left({\upgamma }^{*}+1\right)\right]}{64\left\{{\Psi }_{1}\right\}}>0.$$

## Numerical study

In this section, we conduct a mathematical exploration to compare the performance of our suggested estimator with existing estimators. We consider four actual datasets that are sampled using simple random sampling, and their descriptions are provided in Table 1. To compare the performance of the estimators, we use the concept of percentage relative efficiency (PRE), which is a widely used measure in statistics.

**Table 1:** Summary statistics of population’s I–IV.

Parameters	Populations			
	I	II	III	IV
*N*	80	33	69	34
*N*	24	8	25	10
$${\reflectbox{$\lambda$}}$$	0.02916667	0.09469697	0.02550725	0.7058824
$\overline{Y }$	5182.637	27.49091	4514.899	765.3529
${\overline{X} }_{1}$	285.125	3.727273	4954.435	218.4118
${\overline{X} }_{2}$	1126.463	72.54545	4591.072	201.4118
${S}_{y}^{2}$	3,369,642	102.6327	37,199,578	222,931.4
${S}_{x1}^{2}$	73,132.09	2.329545	49,829,270	28,123.22
${S}_{x2}^{2}$	715,055.8	111.8807	39,881,874	23,154.86
${C}_{y}$	0.3541939	0.3685139	1.350893	0.6169129
${C}_{x1}$	0.9484593	0.4094911	1.424781	0.7678148
${C}_{x2}$	0.7506772	0.1458033	1.375541	0.755503
${\rho }_{yx1}$	0.9149811	0.432738	0.9601401	0.8307546
${\rho }_{yx2}$	0.9413055	0.2521603	0.956907	0.8992855
${\rho }_{x1x2}$	0.9884207	− 0.06598959	0.972902	0.929922
${{\mkern0.75mu\mathchar '26\mkern -9.75mu\lambda}}_{400}^{*}$	1.210342	4.382758	6.544991	1.583558
${{\mkern0.75mu\mathchar '26\mkern -9.75mu\lambda}}_{040}^{*}$	2.491817	1.245254	8.69773	2.091393
${{\mkern0.75mu\mathchar '26\mkern -9.75mu\lambda}}_{004}^{*}$	1.79522	1.015036	8.698631	2.341373
${{\mkern0.75mu\mathchar '26\mkern -9.75mu\lambda}}_{220}^{*}$	1.294326	0.388943	7.053057	1.004473
${{\mkern0.75mu\mathchar '26\mkern -9.75mu\lambda}}_{202}^{*}$	1.193045	1.218625	7.190267	1.607816
${{\mkern0.75mu\mathchar '26\mkern -9.75mu\lambda}}_{022}^{*}$	2.093142	0.4465917	8.523088	1.433162

To calculate the PRE, we use the following expression:

$$\mathrm{PRE}(.) = \frac{Var\left( {\mathrm{T}}_{u}\right)}{MSE\left({\mathrm{T}}_{prop}\right)} \times 100$$

where (*u*) = (0, 1, 2, 3, 4, 5, 6).

The expression of mean square error (MSE) using four actual data sets are given in Table 2. The conditional values of estimators are given in Table 3. The expression of percentage relative efficiency (PRE) using four actual data sets are given in Table 4.

**Table 2:** Mean square error using population’s I-IV.

Estimators	Data-I	Data-II	Data-III	Data-IV
${\mathrm{T}}_{0}$	400,831,931,440	4371.75	231,019,500,000,000	5,555,316,462
${\mathrm{T}}_{1}$	368,762,720,252	4837.944	40,118,900,000,000	5,844,551,874
${\mathrm{T}}_{2}$	178,180,420,058	4250.572	29,141,750,000,000	3,862,867,615
${\mathrm{T}}_{3}$	175,427,522,009	3028.487	28,540,710,000,000	3,584,275,528
${\mathrm{T}}_{4}$	261,285,129,365	4543.687	67,642,260,000,000	4,742,748,835
${\mathrm{T}}_{5}$	923,630,298,554	6538.664	138,236,600,000,000	11,758,993,996
${\mathrm{T}}_{6}$	171,317,454,680	2937.575	25,915,450,000,000	3,436,287,618
${\mathrm{T}}_{prop}$	75,030,307,291	2200.201	18,697,750,000,000	1,549,496,322

**Table 3:** Conditional values using actual data sets.

Conditional values	Data-I	Data-II	Data-III	Data-IV
1	325,801,624,149	2171.549	212,321,800,000,000	4,005,820,140
2	293,732,412,961	2637.743	21,421,150,000,000	4,295,055,552
3	103,150,112,767	2050.371	10,444,000,000,000	2,313,371,293
4	100,397,214,718	828.286	9,842,960,000,000	2,034,779,206
5	186,254,822,074	2343.486	48,944,510,000,000	3,193,252,513
6	848,600,000,000	4338.463	119,538,900,000,000	10,209,497,674
7	96,287,147,389	737.374	7,217,700,000,000	1,886,791,296

**Table 4:** Percentage relative efficiency w.r.t ${\mathrm{T}}_{0}$ of population’s I–IV.

Estimators	Data-I	Data-II	Data-III	Data-IV
${\mathrm{T}}_{0}$	100	100	100	100
${\mathrm{T}}_{1}$	108.6964	90.36379	575.8359	95.0512
${\mathrm{T}}_{2}$	224.9585	102.8508	792.7441	143.8133
${\mathrm{T}}_{3}$	228.4886	144.3542	809.4386	154.9913
${\mathrm{T}}_{4}$	153.4079	96.2159	341.5313	117.1328
${\mathrm{T}}_{5}$	43.39744	66.85998	167.119	47.24313
${\mathrm{T}}_{6}$	233.9703	148.8217	891.4353	161.6662
${\mathrm{T}}_{prop}$	534.2267	198.6978	1235.547	358.524

**Population 1:** [Source: Murthy^27^]

*Y*: Output of the workshop,

$${X}_{1}$$=Fixed capital,

${X}_{2}$= Number of worker.

**Population 2:** [Source: Cochran^28^]

*Y*: Food cost,

${X}_{1}$: size of the family,

and $${X}_{2}$$= Income.

**Population 3:** [Source: Singh^29^]

*Y*: Estimated fish caught in the year 1995,

${X}_{1}$: Estimated fish caught in the year 1994,

${X}_{2}$= Estimated fish caught in the year 1993.

**Population 4: **[Source: Sukhathme^30^]

*Y*: Area under wheat in 1937,

${X}_{1}$= Area under wheat in 1936,

${X}_{2}$= Total cultivated area in 1931.

## Simulation study

This portion shows the algebraic method for the evaluation of T_prop_ with the existing counterparts T_0_, T_1_, T_2_, T_3_, T_4_, T_5,_ and T_6_. The algorithm for simulation study to demonstrate the performance of numerous estimators of T_0_ is as follows:

1. Produce two independent random variables X from N $\left( {\mu ,\sigma^{2} } \right)$ and Z from N $\left( {\mu_{1} ,\sigma_{1}^{2} } \right)$ using the box-Muller method.
2. Set $$Y=\rho X+\sqrt{1-{\rho }^{2}}$$, where $$0<\rho =0.5, 0.6, 0.7, 0.8>1$$.
3. Return the pair (Y, X, Z).
4. Let the population-I with the parameters $\mu = 3,$ $\sigma =2,$ $\mu_{1} = 5$ and $\sigma_{1} = 3$ and in step-1 and repeat steps 1–3 for 500 times. This population will have different variances for X and Z.
5. Likewise, produce the population-II with the parameters $\mu =2, \sigma =3,$ ${\mu }_{1}=4$ and $\sigma_{1} = 3$ in step-1 and repeat steps 1–3 for 500 times.
6. From the population of size N = 50, draw 500 SRSWOR (${y}_{i}$,${x}_{i}$) (i = 1, 2,3,…,n) of size n = 10, 15 and 20.
7. The Average MSE of the estimators is defined by:
  $$Average MSE\left(T\right)=\frac{1}{500}\sum_{k=1}^{500}E{({T}_{k}-\mathrm{T}0)}^{2}$$
8. The percentage relative efficiency of estimators as compared to usual estimator *T*_*0*_ is defined by:
  $$PRE\left(T\right)=\frac{Var(\mathrm{T}0)\times 100}{MSE(T)}$$
9. The Average PRE of the estimators is defined by
  $$Average PRE\left(T\right)=\frac{1}{500}\sum_{k=1}^{500}PRE({T}_{k})$$

The results of the simulation study are presented in Tables 5 and 6.

**Table 5:** Evaluation of T_prop_ with other existing counterparts for Population-I.

$\rho$	N	Average PRE’s							
		T_0_	T_1_	T_2_	T_3_	T_4_	T_5_	T_6_	T_prop_
0.5	10	100.00	47.42	103.86	118.43	68.90	37.88	123.56	124.09
	15	100.00	44.45	101.50	109.32	67.12	36.44	112.12	112.48
	20	100.00	45.74	102.28	106.54	66.64	33.71	107.94	108.64
0.6	10	100.00	42.94	102.18	115.53	65.66	35.89	120.97	121.87
	15	100.00	53.24	102.08	109.84	71.89	37.77	111.77	112.09
	20	100.00	33.66	103.00	106.09	56.56	26.63	107.91	108.02
0.7	10	100.00	18.50	103.24	113.27	42.77	24.99	132.30	133.85
	15	100.00	39.90	101.76	107.87	62.42	31.34	110.52	111.60
	20	100.00	48.88	102.37	106.02	67.43	31.61	107.10	109.01
0.8	10	100.00	29.70	102.52	113.65	55.26	30.24	122.89	123.07
	15	100.00	36.96	101.31	107.49	60.68	31.13	110.59	111.08
	20	100.00	49.21	101.69	105.95	68.80	34.50	107.15	107.55

**Table 6:** Evaluation of T_prop_ with other existing counterparts for Population-II.

$\rho$	N	Average PRE’s							
		T_0_	T_1_	T_2_	T_3_	T_4_	T_5_	T_6_	T_prop_
0.5	10	100.00	33.49	103.55	112.82	56.57	27.87	118.74	119.25
	15	100.00	38.63	101.48	105.76	58.40	25.35	107.66	110.68
	20	100.00	38.80	102.53	105.45	59.93	26.57	106.80	107.81
0.6	10	100.00	34.59	101.65	110.67	57.33	27.82	115.92	116.16
	15	100.00	47.10	102.33	111.01	68.85	38.40	113.77	114.45
	20	100.00	42.67	103.10	106.61	63.67	30.06	107.98	108.54
0.7	10	100.00	43.50	102.70	112.68	63.78	30.70	116.54	117.09
	15	100.00	41.27	102.24	107.71	62.42	29.77	109.94	110.04
	20	100.00	49.73	103.13	107.11	68.95	33.36	108.30	109.67
0.8	10	100.00	40.37	101.55	110.84	60.96	29.07	114.79	115.00
	15	100.00	41.47	103.11	109.56	63.87	32.42	112.27	113.43
	20	100.00	55.97	103.38	107.88	72.28	36.05	108.90	111.05

## Discussion

As stated before we take four data sets and simulation, to notice the performance of the proposed enhanced variance estimator based on simple random sampling using two auxiliary variables. The proposed estimator is compared with existing estimators concerning MSE and PRE. The summary statistic of actual data sets is presented in Table 1. The MSE of estimators are given in Table 2 and the conditional values of estimators are given in Table 3. From the mathematical results using actual data, and PRE are given in Table 4, it is observed that the proposed estimator is efficient in terms of efficiency. We evaluated the efficiency of our proposed variance estimator in simple random sampling using a simulation study. From Tables 5 and 6, it can be concluded that for different values of the correlation coefficient $\rho$ = 0.5, 0.6, 0.7, 0.8 and the sample size n = 10, 15, 20 the performance of proposed estimator *T*_*prop*_ is more efficient in comparison with the usual estimator and other existing estimators. The result of the simulation study clearly shows that the average PRE of the proposed estimator *T*_*prop*_ is higher as compared to the average PRE of the existing estimator for both the simulated data sets I and dataset II. Hence it can be recommended to use it further.

## Conclusion

In this article, we have suggested a novel variance estimator using two auxiliary variables under simple random sampling. We conducted a comprehensive comparison of our suggested estimator with several existing counterparts using four real data sets. We also obtained the properties of the proposed estimator under the first order of approximation and carried out a simulation study to test its robustness and generalizability. The results showed that the suggested estimator outperformed to the existing estimators in terms of efficiency. Future research could focus on extending our proposed estimator to two-phase sampling designs, and to situations with non-response and measurement error, where information from auxiliary variables can be utilized to estimate population variance more accurately. Additionally, it would be interesting to investigate the performance of our proposed estimator in more complex survey settings, such as clustered and stratified sampling.
