# Author Correction: An enhanced estimator of finite population variance using two auxiliary variables under simple random sampling

**DOI:** 10.1038/s41598-024-57145-4

**Published:** 2024-04-02

**Authors:** Sohaib Ahmad, Nitesh Kumar Adichwal, Muhammad Aamir, Javid Shabbir, Najwan Alsadat, Mohammed Elgarhy, Hijaz Ahmad

**Affiliations:** 1 Department of Statistics, Abdul Wali Khan University Mardan, Mardan, Pakistan; 2 School of Business, University of Petroleum and Energy Studies, Dehradun, India; 3 Department of Statistics, University of Wah at Wah Cant, Wah, Pakistan; 4 Department of Quantitative Analysis, College of Business Administration, King Saud University, P.O. Box 71115, 11587 Riyadh, Saudi Arabia; 5 Mathematics and Computer Science Department, Faculty of Science, Beni-Suef University, Beni‑Suef, 62521 Egypt; 6 Section of Mathematics, International Telematic University Uninettuno, Corso Vittorio Emanuele II, 39, 00186 Rome, Italy; 7 Operational Research Center in Healthcare, Near East University, TRNC Mersin 10, Nicosia, 99138 Turkey; 8 Center for Applied Mathematics and Bioinformatics, Gulf University for Science and Technology, Mishref, Kuwait; 9 Department of Computer Science and Mathematics, Lebanese American University, Beirut, Lebanon

Correction to:* Scientific Reports* 10.1038/s41598-023-44169-5, published online 05 December 2023

The original version of this Article omitted affiliations for Hijaz Ahmad. The correct affiliations are listed below

Section of Mathematics, International Telematic University Uninettuno, Corso Vittorio Emanuele II, 39, 00186 Rome, Italy

Operational Research Center in Healthcare, Near East University, TRNC Mersin 10, Nicosia, 99138, Turkey

Center for Applied Mathematics and Bioinformatics, Gulf University for Science and Technology, Mishref, Kuwait

Department of Computer Science and Mathematics, Lebanese American University, Beirut, Lebanon

The original Article has been corrected.

